# Colorimetric Sensor Based on the Oxidase-like Activity of Au@MnO_x_ Nanozyme for High-Sensitivity Detection of Malathion

**DOI:** 10.3390/foods15213967

**Published:** 2026-11-05

**Authors:** Lu Lin, Xuan Wang, Li-Jiao Wang, Guo-Yuan Xiong

**Affiliations:** 1 College of Food Science and Engineering, Anhui Science and Technology University, Chuzhou 239000, China; 2 Anhui Province Key Laboratory of Functional Agriculture and Functional Food, Anhui Science and Technology University, Chuzhou 239000, China

**Keywords:** Au@MnO_x_, oxidase-like activity, colorimetric, malathion, food safety

## Abstract

Organophosphorus pesticide residues severely threaten agricultural products. Thus, it is important to explore sensitive and efficient analytical detection methods for target detection. In this study, a lychee-like gold core-manganese oxide shell structure (Au@MnO_x_) was synthesized, which displays prominent oxidase (OXD)-like activity. It is able to catalyze 3,3′,5,5′-tetramethylbenzidine (TMB) oxidation to a blue product (oxTMB) without the need for hydrogen peroxide. Malathion can inhibit acid phosphatase (ACP) activity, reduce L-ascorbic acid (AA) production, weaken the color-quenching effect, and restore blue color to the system. Based on this “signal inhibition-recovery” mode, a colorimetric sensor was constructed and applied to quantitatively analyze malathion in food matrices, with a limit of detection (LOD) of 1.51 ng/mL. The average recoveries of malathion in cucumber and cabbage samples were in the ranges of 97.2–104.5% and 99.4–102.5%, respectively, with all relative standard deviations (RSDs) less than 7%. This study provides a sensitive and efficient colorimetric detection strategy for malathion, which has significant application value in the field of food safety.

## 1. Introduction

As a widely used compound worldwide [1,2], organophosphate pesticides (OPs) are essential for controlling crop diseases, insect pests, and weeds [3,4]. However, the actual production and extensive use of OPs are not carried out in accordance with the regulations, resulting in excessive residues being found in agricultural products and the ecological environment [5,6]. More seriously, once these residual pesticides enter an organism through the food chain [7], they will harm the human immune system and endocrine system and can even cause cancer. In addition, OPs can irreversibly inhibit cholinesterasethe activity, which causes acetylcholine to accumulate in the body [8,9]. Meanwhile, they impair the normal regulatory function of the nervous system and bring serious risks to human health [10,11]. Moreover, the harmful components of pesticides entering water, soil, and other environments will also cause pollution of nature, destroy the ecological balance, and affect biodiversity. Developing sensitive, reliable, and rapid detection approaches for pesticide residues is critical to guarantee food safety and maintain the ecological environment.

Traditional instrument-based detection methods for pesticide residues have high precision and accuracy [12,13,14]. However, large instruments and equipment are expensive, have high maintenance costs, long testing cycles, and require complex sample pretreatment processes that require professional operators, making it hard to carry out on-site detection at the grassroot level [15,16]. Nanoenzymes are a type of enzyme mimic that can exhibit catalytic performances analogous to natural enzymes at the nanoscale, and they are essentially nanomaterials [17,18,19]. In recent years, nanoenzymes have shown extensive application potential in biomedical detection, agricultural product safety, environmental monitoring, and other areas due to their high stability, strong compatibility, and ability to simulate various enzyme activities [20,21,22,23]. As a fast and portable detection method, the colorimetric method features intuitive visible results and is well suited for field on-site detection [24]. The combination of nanoenzymes and colorimetric methods not only presents excellent potential for analytical and monitoring research but also gives rise to a series of high-performance nanoenzyme colorimetric sensing strategies [25,26]. Compared with traditional methods, the nanozyme-based colorimetric method is usually simple to operate, fast in response, and low in cost [27]. It does not require large-scale equipment, which is extremely conducive to the development of portable sensor devices [28,29,30]. In addition, nanozymes have excellent stability and are easy to synthesize and store on a large scale [31,32]. These advantages significantly enhance the reliability and service life of sensors. Therefore, exploring the application of nanozymes in colorimetric sensing is of great significance and research value.

**Scheme 1:**
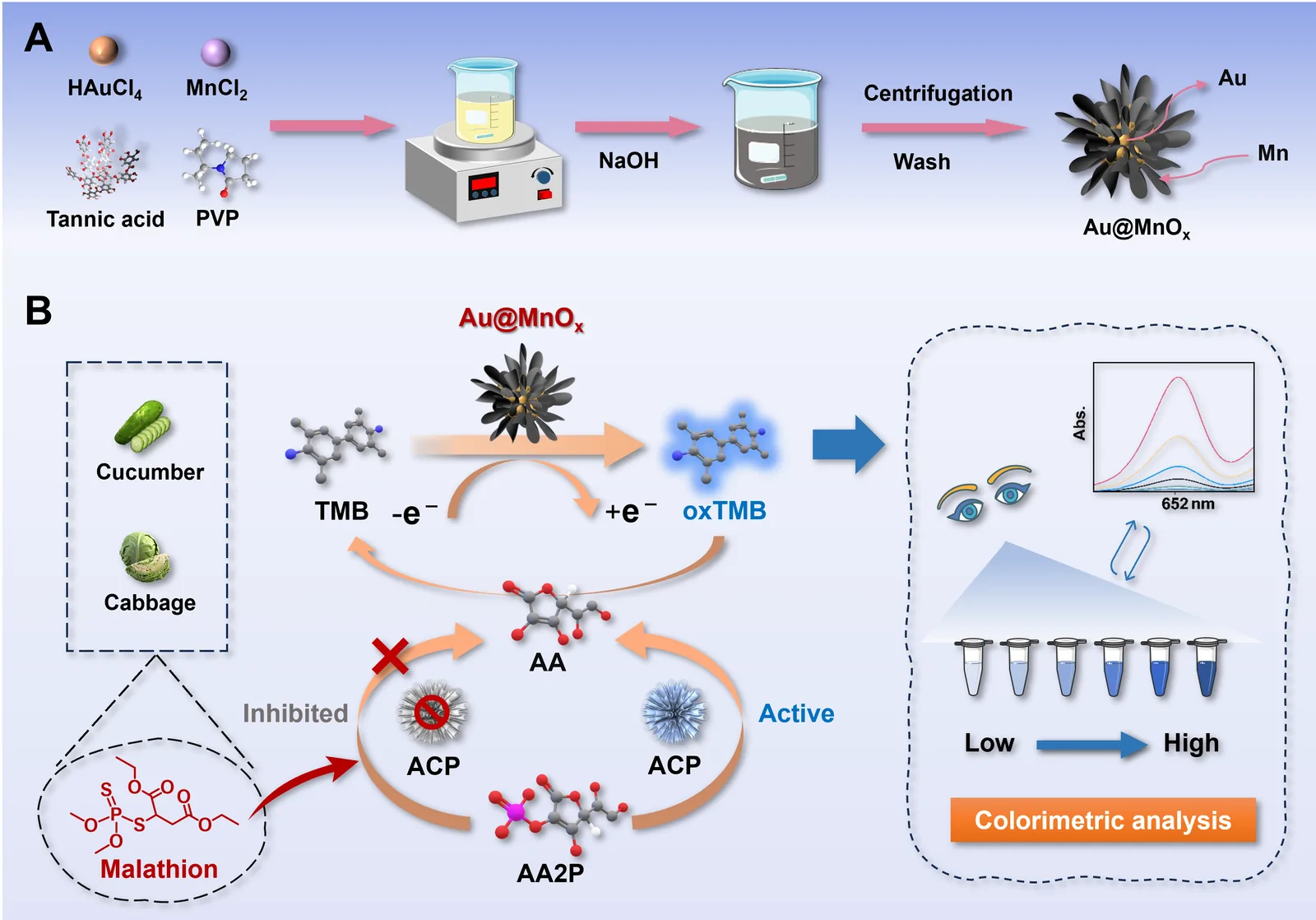
Schematic diagram of (**A**) synthesis of Au@MnO_x_ nanoenzyme and (**B**) the construction of a colorimetric probe for malathion residue detection based on the Au@MnO_x_ nanozyme.

In this study, a lychee-like gold core-manganese shell structure (Au@MnO_x_) has been synthesized (Scheme 1A). Subsequently, a colorimetric sensing platform using Au@MnO_x_ was established (Scheme 1B). The Au@MnO_x_ nanozyme exhibits excellent oxidase (OXD)-like activity. It can catalyze the oxidation of 3,3′,5,5′-tetramethylbenzidine (TMB) to form blue oxidized TMB (oxTMB) through electron transfer, without the participation of hydrogen peroxide. L-ascorbic acid-2-phosphate (AA2P) can be hydrolyzed by acid phosphatase (ACP) to yield ascorbic acid (AA). AA can effectively reduce oxTMB and compete with nanoenzyme activity, resulting in color fading of the solution. When malathion is present in the system, ACP activity can be suppressed, which reduces the yield of AA. This process further weakens the quenching effect of the color reaction, allowing the system to restore a blue color. Based on this, the colorimetric sensing platform was established to detect residues of malathion.

## 2. Materials and Methods

### 2.1. Apparatus and Reagents

The micromorphology of Au@MnO_x_ was analyzed by transmission electron microscopy (TEM) (FEI Talos F200x, Thermo Fisher Scientific, Waltham, MA, USA). The surface elemental composition and chemical valence state of Au@MnO_x_ were analyzed using X-ray photoelectron spectroscopy (XPS) (ESCALAB 250Xi, Thermo Fisher Scientific, Waltham, MA, USA). X-ray diffraction (XRD) (D8 ADVANCE, Bruker, Karlsruhe, Germany) was used to characterize the crystal structure of nanozymes. Fourier transform infrared spectroscopy (FTIR) (Nicolet IS10, Thermo Fisher Scientific, Waltham, MA, USA) was used to characterize the functional groups and chemical bond information on the Au@MnO_x_ surface. Ultraviolet-visible spectroscopy was used to measure UV–visible (UV-vis) spectra using a spectrophotometer (UV-1800, Shimadzu, Kyoto, Japan).

Gold chloride trihydrate (HAuCl_4_·3H_2_O), manganese chloride (MnCl_2_·4H_2_O), tartaric acid (TA), polyvinyl pyrrolidone (PVP, MW~10,000), sodium hydroxide (NaOH), sodium tetraborate decahydrate, boric acid, 3,3′,5,5′-tetramethylbenzidine (TMB), 2,2′-azinobis(3-ethylbenzthiazoline-6-sulfonic acid) (ABTS), o-phenylenediamine (OPD), benzoquinone (BQ), L-Histidine (L-His), isopropanol (IPA), ethylenediaminetetraacetic acid disodium salt (EDTA-2Na), L-ascorbic acid 2-phosphate (AA2P), and acid phosphatase (ACP) were purchased from Aladdin Biochemical Technology Co., Ltd. (Shanghai, China). Malathion, triazophos (TZP), phorate (PHO), isofenphos-methyl (IFM), parathion-methyl (PRM), parathion (PRT), and dicofol (DIC) were acquired from TMRM Quality Inspection Technology Co., Ltd. (Changzhou, China).

### 2.2. Synthesis of Au@MnO_x_

Au@MnO_x_ was synthesized by following a previously documented procedure with minor modifications [33]. Briefly, HAuCl_4_·3H_2_O solution (1%, *w*/*v*) was added to 15 mL of ultrapure water under continuous stirring until the mixture was well mixed. After that, MnCl_2_·4H_2_O solution (100 mM), tartaric acid (TA, 0.58 mM), and polyvinylpyrrolidone (PVP, 1 mM, MW 10,000) were added to the mixture in sequence with constant stirring. Thereafter, NaOH solution (100 mM) was introduced into the mixture. Immediately upon addition, the solution gradually turned from pale yellow into grayish-black. Under constant stirring, the mixture was maintained at room temperature for 30 min. After the reaction was complete, the obtained product was separated and collected by centrifugation at 5000× *g* for 20 min. Then the supernatant was discarded, and the resultant product was washed and purified with ultrapure water three times to remove the remaining reactants, ensuring that the product was evenly dispersed during washing. Finally, the washed product was dispersed in boric acid buffer (200 mM, pH 8.0) for later use.

### 2.3. OXD-like Activity of Au@MnO_x_

The OXD-like performance of Au@MnO_x_ was measured via the steady-state dynamics method. In the reaction system, the concentration of Au@MnO_x_ remains constant, and TMB solutions of different concentrations are added separately. After rapid mixing, the reaction system absorbance at a wavelength of 652 nm was immediately monitored over time with a UV–visible (UV-vis) spectrophotometer. The initial reaction rate (*v*) was measured based on the amount of oxidized TMB (oxTMB) produced per unit time. Specifically, the value of *v* was derived from the absorbance change rate at 652 nm and the corresponding molar extinction coefficient of oxTMB at this wavelength, in accordance with the Lambert–Beer law. The values of kinetic parameters *K*_m_ and *V*_max_ were determined by fitting *v* against substrate concentration ([*S*]) according to the Michaelis–Menten equation as follows (Equation (1)):

(1) $$v=\frac{V_{\max}[S]}{K_{m}+[S]}$$

To obtain linear fitting results and facilitate accurate calculation of the kinetic parameters, the experimental data were further processed by using the Lineweaver–Burk double reciprocal plotting approach (2) to calculate *V*_max_ and *K*_m_:

(2) $$\frac{1}{v}=\frac{K_{m}}{V_{\max}}(\frac{1}{\left[S\right]}+\frac{1}{K_{m}})$$

### 2.4. Colorimetric Detection of Malathion

The experimental procedure for colorimetric determination of malathion was performed as follows. Briefly, 10 μL ACP solution (40 U/L) and serial concentrations of malathion solution (50 μL) were thoroughly mixed with 700 μL of 100 mM NaAc-HAc buffer (pH 4.0). Then, the resulting mixture was vortexed until homogeneous and then incubated at 37 °C for 20 min. After the incubation process was finished, the reaction system was supplemented with 10 μL AA2P solution (6 mM), 70 μL Au@MnO_x_ (100 μg/mL), and 300 μL TMB solution (3 mM). The reaction was further continued for another 20 min at 37 °C to ensure sufficient reaction. Finally, UV-vis spectral scanning was performed to acquire the absorption profiles of the reaction mixtures.

### 2.5. Pretreatment of Actual Samples

To confirm whether the proposed colorimetric method is feasible, this study selected cucumber and cabbage for malathion spiked recovery experiments. Fresh cucumbers and cabbages were obtained from a supermarket in Chuzhou. In the spiked recovery experiment, the spiked concentrations of samples were 0.1, 0.5, and 2.0 μg/g, respectively. The specific preprocessing process for the sample was as follows. First, the edible portion of each sample was homogenized with a tissue homogenizer at 12,000 rpm for 2 min. Subsequently, 10.0 g of the homogenized sample was accurately weighed and transferred into a 50 mL polytetrafluoroethylene centrifuge tube. After adding 20 mL of acetonitrile, the mixture was vortexed at 2500 rpm for 2 min. Then, 0.1 g of activated carbon powder was added, and vortexing was continued for another 1 min to fully adsorb pigments from the sample. The mixture was then centrifuged at 4500× *g* for 10 min at room temperature. The supernatant was carefully collected and filtered through a 0.22 μm hydrophilic polyethersulfone (PES) syringe filter. An aliquot of 0.5 mL filtrate was taken and dried under nitrogen flow at 40 °C. Finally, the residue was reconstituted with 0.5 mL of 100 mM NaAc-HAc buffer (pH 4.0), and the reconstituted solution was subjected to the subsequent colorimetric assay.

## 3. Results

### 3.1. Synthesis and Characterization of Au@MnO_x_

As illustrated in Scheme 1A, the Au@MnO_x_ with lychee-like structures were prepared by one-step co-reduction and co-precipitation methods. In this synthetic system, TA served as a critical reductant, while PVP functioned as a steric stabilizer to prevent agglomeration of the as-synthesized nanocomposites. Specifically, NaOH solution was introduced to rapidly raise the system pH to the alkaline range. Under such alkaline conditions, TA efficiently reduced Au^3+^ ions to Au atoms, which further aggregated to form cores. Simultaneously, Mn^2+^ ions were oxidized and precipitated in the alkaline and oxidizing atmosphere, with the Au acting as heterogeneous nucleation sites. During material synthesis, the reaction system undergoes a rapid color change from pale yellow to grayish black, which may be due to the generation of gold nanoparticles and the encapsulation of manganese oxide shells.

**Figure 1:**
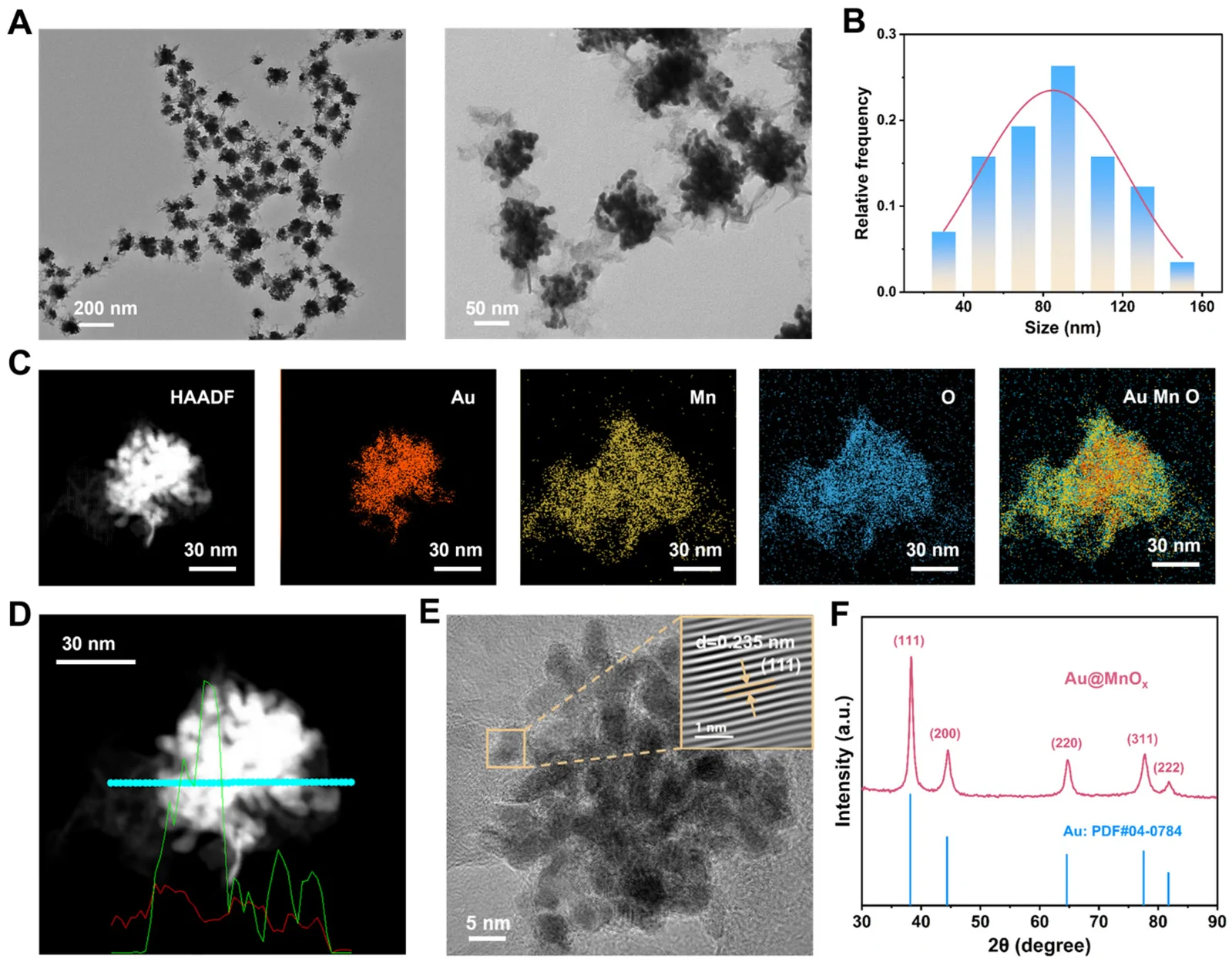
The characterization of Au@MnO_x_. (**A**) TEM images of Au@MnO_x_ (Left: low magnification; Right: high magnification). (**B**) Particle size distribution histogram of Au@MnO_x_. (**C**) HAADF-STEM image and corresponding EDS elemental mapping of Au@MnO_x_. (**D**) EDS line scan profile of a single Au@MnO_x_. The cyan line denotes the scanning track, while the green and red curves represent the line signals of Au and Mn elements, respectively. (**E**) High-resolution TEM (HR-TEM) image of Au@MnO_x_ (Inset: lattice fringes of the Au (111) plane). (**F**) XRD pattern of Au@MnO_x_.

Transmission electron microscopy (TEM) was adopted to characterize the structural and morphological features of Au@MnO_x_. As shown in Figure 1A, Au@MnO_x_ exhibited good dispersibility and uniform size, and individual particles can be observed to have a distinct lychee-like morphology and clear core–shell nanostructure. The particle size distribution histogram (Figure 1B) showed a mean particle diameter of 86 nm. Elemental distribution was characterized by means of high-angle annular dark field scanning transmission electron microscopy (HAADF-STEM) coupled with energy-dispersive spectroscopy (EDS). The EDS element mapping (Figure 1C) confirms the formation of the Au core and the manganese oxide (MnO_x_) shell. This result was further validated by the EDS line scan spectrum (Figure 1D). Quantitative EDS analysis (Figure S1) showed that the atomic percentages (At%) of O, Mn, and Au in the sample were 38.86%, 31.35%, and 29.79%, respectively, confirming the presence of all target elements in the Au@MnO_x_.

HR-TEM imaging (Figure 1E) displayed clear lattice fringes, and the measured lattice stripe spacing was 0.235 nm, which matched well with the (111) crystal plane of metallic Au [34,35]. This result was further validated by using XRD characterization. As presented in Figure 1F, the characteristic diffraction peaks located at 38.2°, 44.4°, 64.6°, and 77.6° were attributed to the (111), (200), (220), and (311) crystal planes of face-centered cubic (FCC) metallic Au (JCPDS No. 04-0784) [36,37,38], respectively, demonstrating the high crystallinity of the Au component in the composite material [39]. It is worth noting that no characteristic diffraction peaks corresponding to MnO_x_ were detected in the XRD pattern, indicating that the MnO_x_ component may exist in an amorphous state. These structural features enable sufficient exposure of active sites, thereby significantly improving the catalytic properties of Au@MnO_x_.

**Figure 2:**
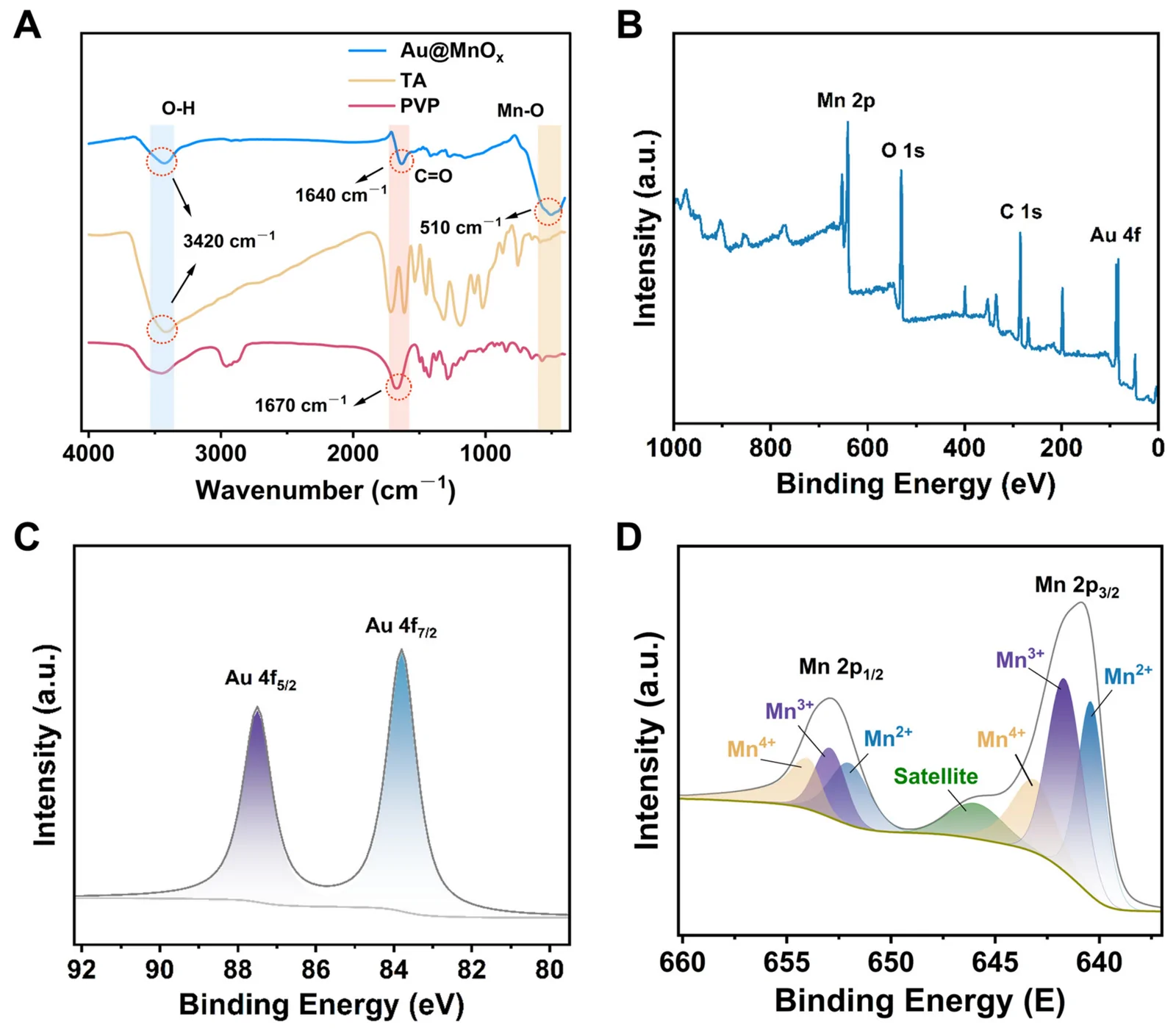
Structural and elemental characterizations of Au@MnO_x_. (**A**) FTIR spectra of Au@MnO_x_, pure TA, and PVP. (**B**) XPS full-survey spectrum of Au@MnO_x_. High-resolution XPS spectra of (**C**) Au 4f and (**D**) Mn 2p.

FTIR was used to further investigate the surface chemical groups of Au@MnO_x_ (Figure 2A). The broad absorption signal appearing at around 510 cm^−1^ is attributed to the characteristic stretching vibration of the Mn-O bond, indicating the presence of MnO_x_. Combined with XRD analysis results, MnO_x_ may mainly exist in an amorphous state. The absorption band around 1640 cm^−1^ originates from the C=O stretching vibration of PVP. Compared with pure PVP, the characteristic C=O absorption peak of PVP in Au@MnO_x_ shifts significantly towards lower wave numbers, indicating the interaction between PVP and nanocomposite materials. In addition, the broad hydroxyl stretching vibration band at approximately 3400 cm^−1^ provides evidence for the presence of TA in Au@MnO_x_.

Next, XPS was utilized for the elemental composition analysis of Au@MnO_x_. The results verified that the material mainly consists of Mn, Au, O, and C elements (Figure 2B). Furthermore, the high-resolution Au 4f energy spectrum (Figure 2C) exhibited typical double peaks of spin–orbit splitting located at 83.6 eV and 87.5 eV, which were assigned to Au 4f_7/2_ and Au 4f_5/2_ [40,41], respectively. The obtained value matches well with the characteristic splitting energy of metallic Au, indicating the presence of metallic Au in Au@MnO_x_. Both the Au 4f_7/2_ and Au 4f_5/2_ spectra present as single symmetric peaks, without the characteristic signal of high-valence Au^3+^, demonstrating that Au^3+^ ions from HAuCl_4_ were completely reduced to metallic Au^0^ during the synthesis process [42,43]. The high-resolution Mn 2p spectrum (Figure 2D) displayed typical spin–orbit splitting features, where Mn 2p_3/2_ and Mn 2p_1/2_ were clearly distinguished with a peak separation of approximately 11 eV [44,45], which is consistent with the characteristic spin–orbit splitting of manganese oxides. Peak fitting of the Mn 2p_3/2_ and Mn 2p_1/2_ regions further revealed that the Mn species on the surface of Au@MnO_x_ coexist in three valence states, namely Mn^2+^, Mn^3+^, and Mn^4+^.

### 3.2. Catalytic Activity and Kinetics of Au@MnO_x_

**Figure 3:**
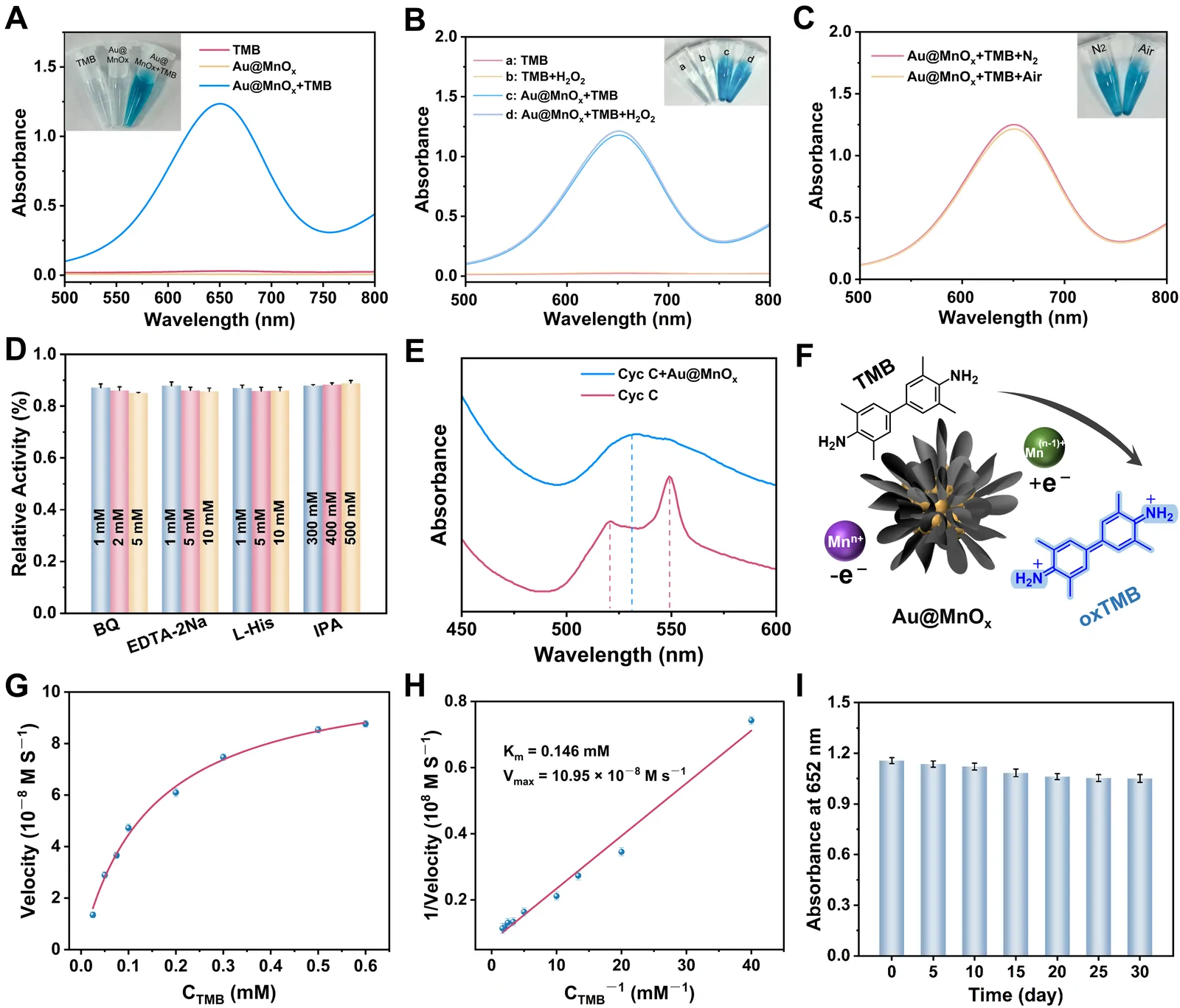
Catalytic activity and mechanism of Au@MnO_x_. (**A**) UV-Vis absorption spectra of different reaction systems. Experimental conditions: pH 4.0 HAc-NaAc buffer, 3 mM TMB, 100 μg/mL Au@MnO_x_. (**B**) Effect of H_2_O_2_ on the TMB oxidation catalyzed by Au@MnO_x_. (**C**) Oxygen dependence of the catalytic reaction confirmed by deoxygenation experiments. (**D**) Influence of various reactive oxygen species (ROS) scavengers on the catalytic activity of Au@MnO_x_. (**E**) UV-Vis absorption spectra of Cyt C in the absence and presence of Au@MnO_x_. (**F**) Schematic illustration of the catalytic mechanism for TMB oxidation via the direct electron transfer pathway mediated by Au@MnO_x_. (**G**) Steady-state enzymatic kinetics of Au@MnO_x_ towards the TMB substrate. (**H**) Lineweaver–Burk plot for the catalytic reaction of Au@MnO_x_. (**I**) Storage stability assessment of Au@MnO_x_.

To screen the optimal substrate, cross-validation was performed with OPD and ABTS serving as auxiliary substrates. The results (Figure S2) demonstrate that the reaction system of Au@MnO_x_ with TMB displays a distinct absorption peak centered at 652 nm, and the inset exhibits an obvious blue color, which is visually observable. Therefore, TMB was identified as the optimal catalytic substrate. The UV-vis absorption spectra for different reaction systems are displayed in Figure 3A. The experimental group with TMB catalyzed by Au@MnO_x_ exhibited a distinct characteristic absorption peak at 652 nm, along with a distinct blue color visible to the naked eye, confirming that the Au@MnO_x_ possessed efficient catalytic activity toward TMB oxidation.

To further clarify the intrinsic enzyme-like catalytic type of Au@MnO_x_, H_2_O_2_-dependent experiments and deoxygenation experiments were conducted. When Au@MnO_x_ and TMB were added, a prominent absorption peak appeared at 652 nm (curve c), which can be assigned to the characteristic signal of oxTMB. As illustrated in the inset of Figure 3B, the reaction system exhibited a distinct blue color. Upon further introduction of H_2_O_2_ into the above system, the shape and intensity of the absorption peak were almost identical to those of curve c. This phenomenon suggests that H_2_O_2_ made no additional contribution to the catalytic reaction. In the deoxygenation experiment, after introducing nitrogen for deoxygenation, the system still showed a clear absorption peak at 652 nm (Figure 3C). This indicates that the reaction of Au@MnO_x_ that catalyzed the oxidation of TMB is independent of O_2_, and the catalytic oxidation ability may stem from the inherent redox properties of the Au@MnO_x_ itself.

### 3.3. Catalytic Mechanism and Performance of Au@MnO_x_

To clarify the underlying catalytic mechanism of Au@MnO_x_ in the TMB color reaction, reactive oxygen species (ROS) scavenging experiments were conducted. Specifically, benzoquinone (BQ), ethylenediaminetetraacetic acid (EDTA-2Na), L-histidine (L-His), and isopropanol (IPA) were used to scavenge singlet oxygen (^1^O_2_), holes, superoxide anion radicals (O_2_^·−^), and hydroxyl radicals (·OH), respectively. Under different concentration gradients, none of the four tested scavengers exhibited inhibition of the catalytic activity of Au@MnO_x_ (Figure 3D). This result suggested that Au@MnO_x_-catalyzed TMB oxidation may originate from non-radical pathways. We further used cytochrome C (Cyt C) as the electron acceptor probe to investigate the electron transfer ability of Au@MnO_x_. The results showed that the system containing only Cyt C exhibited characteristic absorption peaks in the reduced state at 520 nm and 550 nm (Figure 3E) [46,47]. Upon the addition of Au@MnO_x_, the reaction system displayed a 530 nm characteristic absorption signal of oxidized Cyt C, while the characteristic signals at 520 nm and 550 nm exhibited obvious attenuation. This phenomenon indicates that Au@MnO_x_ can accept electrons from Cyt C through direct electron transfer. In summary, the catalytic oxidation of TMB by Au@MnO_x_ is achieved through direct electron transfer driven by the material’s native redox performance. We propose that the catalytic oxidation of TMB likely follows a direct electron transfer pathway (Figure 3F), with multivalent manganese species in Au@MnO_x_ involved in the electron transfer.

Furthermore, critical experimental parameters, including reaction buffer, substrate TMB concentration, Au@MnO_x_ dosage, and reaction temperature, were systematically optimized. Firstly, the influence of buffer type on the color development reaction of TMB catalyzed by Au@MnO_x_ was investigated. The results suggested that the maximum absorbance was recorded in the HAc-NaAc buffer (Figure S3). Then, the pH and concentration of the HAc-NaAc buffer were systematically optimized (Figure S4). As indicated in Figure S4A, the absorbance of oxTMB reached its highest at pH 4.0. On this basis, the optimization results of buffer concentration (Figure S4B) showed that the absorbance reached a plateau at an HAc-NaAc concentration of 100 mM. Under the optimal buffer conditions, the concentrations of the TMB substrate and Au@MnO_x_ were optimized and screened. As displayed in Figure S5A, the absorbance signal at 652 nm reached the maximum at 3 mM. The optimization results of Au@MnO_x_ concentration (Figure S5B) showed that the absorption intensity of 652 nm gradually enhanced as the Au@MnO_x_ concentration increased exhibiting concentration-dependent behavior. Finally, the incubation time and temperature of the colorimetric sensing system were optimized and screened. The absorbance signal at 652 nm reached a stable state after 15 min of reaction (Figure S6A). The results in Figure S6B reveal that the absorbance reaches its maximum at 37 °C. Extremely high temperatures can induce a decline in the catalytic activity of Au@MnO_x_. Therefore, 37 °C was confirmed to be the optimal reaction temperature.

To assess the OXD-like catalytic capability of Au@MnO_x_, the steady-state kinetic behavior of its TMB catalytic reaction was systematically studied (Figure 3G). The Lineweaver–Burk double reciprocal plot (Figure 3H) exhibited an excellent linear fitting performance with a correlation coefficient (R^2^) up to 0.998. The calculated *K*_m_ and *V*_max_ values were 0.146 mM and 10.95 × 10^−8^ M·s^−1^, respectively. Compared to most nanozymes documented in prior studies, Au@MnO_x_ exhibits a lower *K*_m_ value [30,47,48,49], indicating stronger substrate affinity, while maintaining a considerable *V*_max_ [50,51], which demonstrates excellent OXD-like catalytic performance (Table S1). In addition, the storage stability of Au@MnO_x_ was also measured (Figure 3I). After 30 days of storage, the TMB catalytic reaction system containing Au@MnO_x_ maintained stable absorbance at a wavelength of 652 nm, indicating that the catalytic activity of nanoenzymes has long-term stability.

### 3.4. Construction of Detection Probe

**Figure 4:**
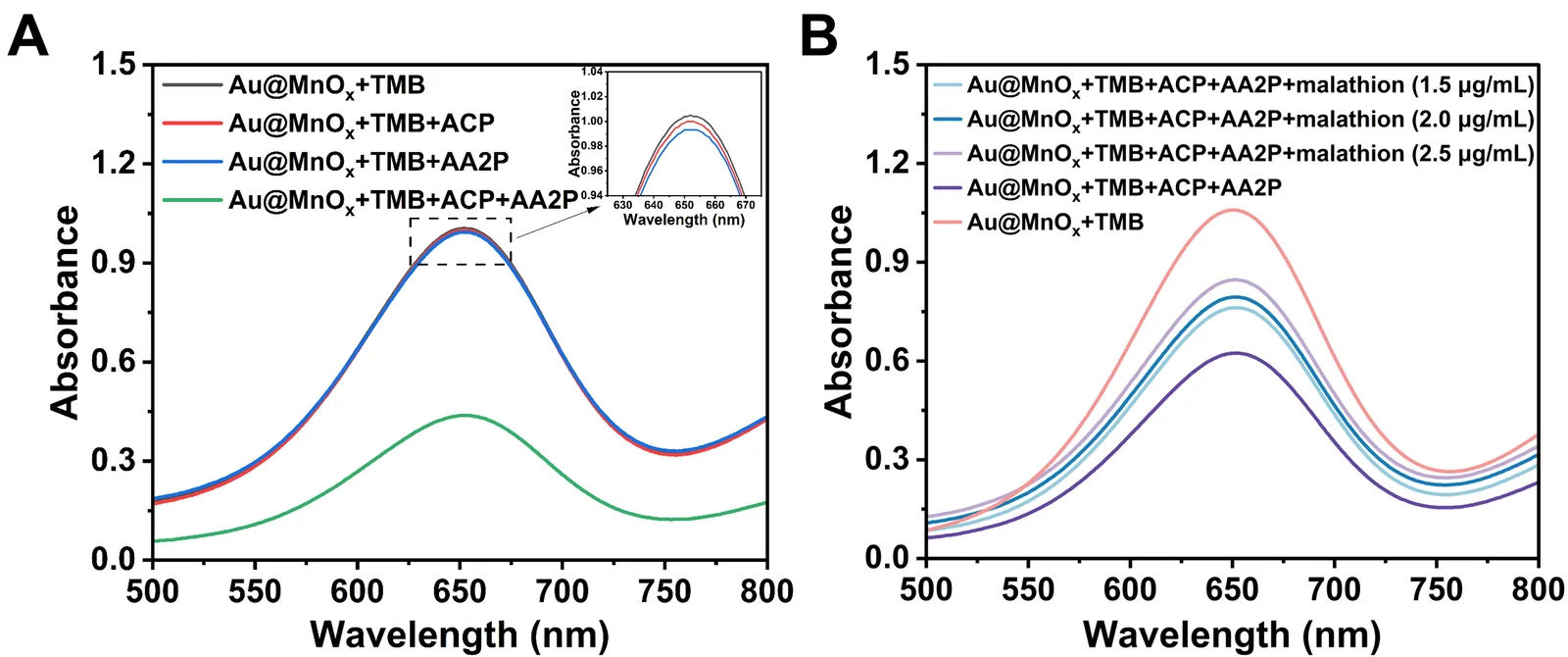
Feasibility verification of the colorimetric assay for malathion detection. UV-vis absorption spectra of (**A**) different reaction systems and (**B**) the sensing system in the presence of varying concentrations (1.5, 2.0, and 2.5 μg/mL) of malathion.

Based on the excellent catalytic OXD-like property of Au@MnO_x_, the colorimetric sensing platform was established for the determination of malathion. As presented in Scheme 1B, Au@MnO_x_ exhibits high catalytic activity toward TMB oxidation to form oxTMB, and the solution system appeared blue and showed an obvious characteristic absorption signal located at 652 nm, which served as the signal output. ACP can specifically hydrolyze substrate AA2P to generate AA with strong reducibility. When ACP and AA2P coexist, a large amount of AA was generated to effectively reduce oxTMB, resulting in blue fading and an obvious reduction at 652 nm, and the signal was quenched (Figure 4A). After adding malathion (Figure 4B), the blue of the system was gradually restored, accompanied by a matched enhancement at 652 nm. The above result demonstrates that malathion is capable of inhibiting ACP activity efficiently, which reduces AA generation and further recovers the color response signal. In addition, increasing the concentration of malathion leads to a slow upward trend at 652 nm. On the basis of the above sensing mechanism, the colorimetric platform can quantitatively determine the concentration of malathion via an absorbance measurement at 652 nm.

### 3.5. Optimization of Probe Detection Conditions

**Figure 5:**
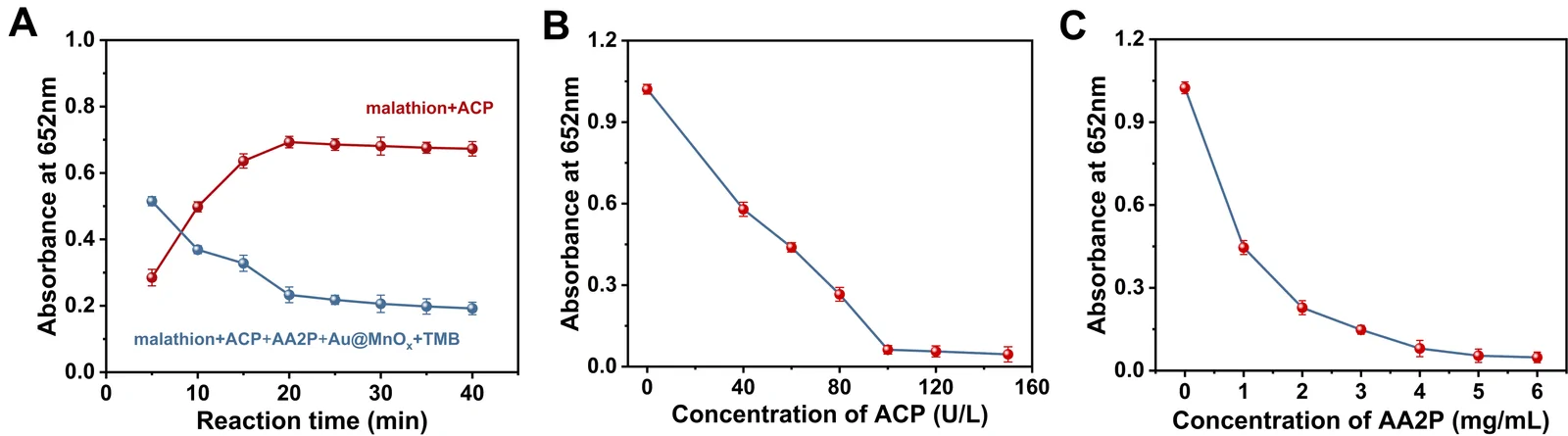
Optimization of key experimental parameters for the colorimetric malathion assay. (**A**) Optimization of the incubation time for malathion with ACP, followed by the subsequent colorimetric reaction optimization, monitored by the absorbance changes at 652 nm. (**B**) Effect of ACP concentration on the absorbance of the sensing system. (**C**) Effect of AA2P concentration on the absorbance of the sensing system. All experiments were performed in 100 mM NaAc-HAc buffer (pH 4.0) at 37 °C.

The key detection parameters that affect colorimetric performance, including reaction time and reagent concentrations, were subjected to systematic optimization. It is shown in Figure 5A that the reaction time for malathion to inhibit ACP activity (red curve) and the TMB colorimetric reaction time (blue curve) were both optimized. This indicated that the inhibition of ACP activity by malathion was basically completed when the reaction lasted for 20 min. Similarly, the competitive reaction between AA and nanoenzymes tended to stabilize at 20 min. Overall, it can be determined that the time for both incubation reactions was 20 min. The AA derived from AA2P hydrolysis by ACP can quench the colorimetric signal. Accordingly, the concentrations of ACP and AA2P were key parameters influencing probe detection. With the increase in ACP concentration, a remarkable reduction in absorbance at 652 nm was observed for the detection system (Figure 5B). As ACP concentration increased to 100 U/L, the absorbance value reached its lowest, indicating that oxTMB was almost completely reduced. Therefore, 100 U/L was confirmed to be the most suitable working concentration of ACP. Likewise, with the increase in AA2P concentration, the absorbance value at 652 nm gradually decreased and reached a steady state at 5 mg/mL, indicating that at this concentration, hydrolysis product AA was enough to completely restore oxTMB and cause the system color to fade away (Figure 5C). All optimization experiments above were performed in three independent replicates (n = 3), and the coefficient of variation (CV) of all measured results was less than 15%, showing acceptable reproducibility.

### 3.6. Analytical Performance of the Probe

**Figure 6:**
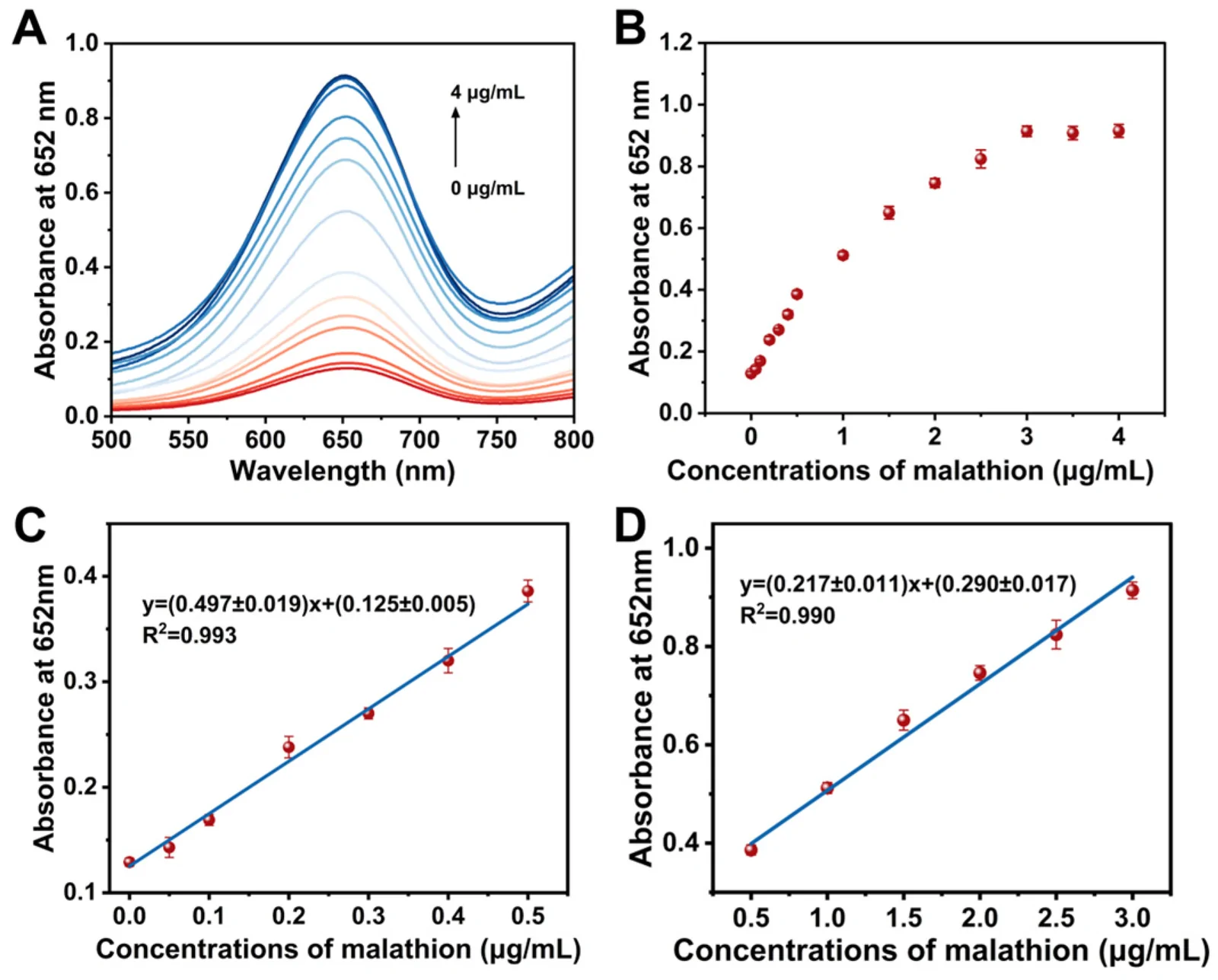
Analytical performance of the developed colorimetric sensor for malathion detection. (**A**) UV-vis absorption spectra of the sensing system with different concentrations of malathion (0–4 μg/mL). (**B**) The corresponding absorbance values at 652 nm as a function of malathion concentration. (**C**,**D**) The linear calibration plots of absorbance at 652 nm versus malathion concentration in the ranges of 0–0.5 μg/mL and 0.5–3.0 μg/mL, respectively.

Under optimized experimental parameters, the sensing capability of the established platform toward malathion was investigated. In Figure 6A, the 652 nm absorption intensity of oxTMB increased with rising malathion concentration. The effect mainly stems from malathion inhibiting ACP activity, resulting in suppressed AA generation and increased oxTMB generation. The scatter plot in Figure 6B further indicated that the absorbance signal at 652 nm correlated linearly and positively with malathion levels. Two distinct linear ranges were identified for the proposed sensor. As shown in Figure 6C, a high-sensitivity linear range was observed for malathion from 0 to 0.5 μg/mL, accompanied by a linear regression fitting equation of y = (0.497 ± 0.019)x + (0.125 ± 0.005) (R^2^ = 0.993). And in Figure 6D, a broader linear range was identified in the concentration range of 0.5–3.0 μg/mL, with a linear fitting equation of y = (0.217 ± 0.011)x + (0.290 ± 0.017) (R^2^ = 0.990). This segmented linear response can be tentatively attributed to the change in the degree of ACP inhibition by malathion. Considering that segmented fitting achieves better linear correlation and most data points match the two calibration curves well, segmented linear calibration was adopted in this study. In the concentration range of 0–0.5 μg/mL, malathion can rapidly inhibit ACP activity, reduce AA production, and weaken the bleaching effect of oxTMB, exhibiting high sensitivity. When the concentration exceeds 0.5 μg/mL, the inhibitory effect on ACP gradually weakens. Further increasing the concentration of malathion can only slightly change the AA yield and absorbance, leading to a decreased slope. According to the 3σ/S method, the limit of detection (LOD) of the developed probe toward malathion was determined to be 1.51 ng/mL, where σ represents the standard deviation of blank sample measurements and S denotes the slope of the calibration curve. This LOD was considerably lower than the statutory maximum residue limits (MRLs) specified in GB 2763-2026 (National Food Safety Standard–Maximum Residue Limits for Pesticides in Food) [52], where the MRLs of malathion in cucumber and cabbage are 0.2 mg/kg and 0.5 mg/kg, respectively. In comparison with other reported probes for OP detection summarized in Table S2, the proposed colorimetric system possesses a wider linear range and a lower LOD [5,53,54,55,56]. Therefore, the developed Au@MnO_x_-based probe demonstrates excellent detection sensitivity toward malathion and is fully competent for practical detection demands.

### 3.7. Specificity and Anti-Interference Ability

**Figure 7:**
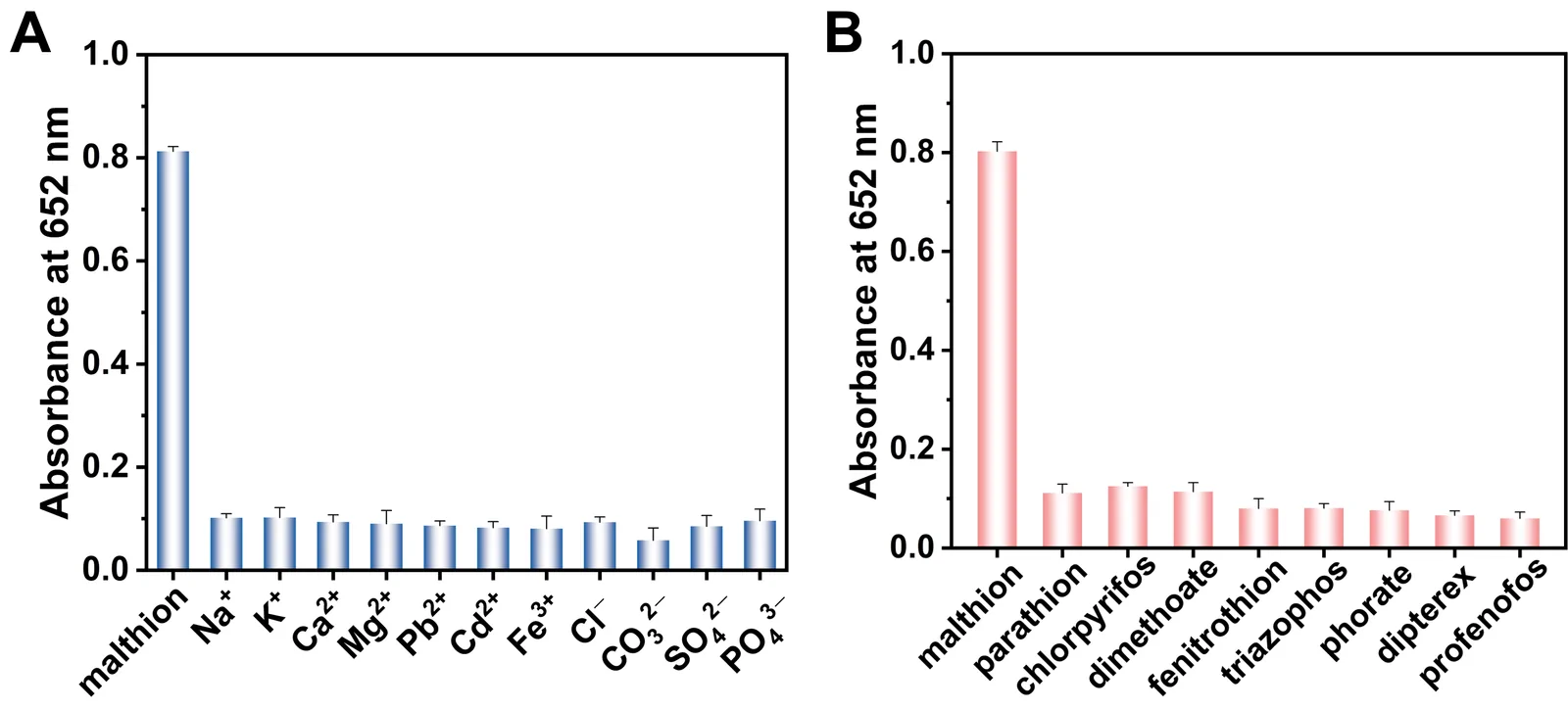
Specificity and anti-interference performance of the malathion colorimetric sensing platform. (**A**) Influence of coexisting ions and (**B**) selectivity against other OPs on the absorbance at 652 nm. Malathion: 2 μg/mL; interfering substances: 10 μg/mL; n = 3.

The accuracy and reliability of the detection approach depend on both its specificity and anti-interference performance. Therefore, the specificity and anti-interference capability of the proposed probe were systematically assessed by introducing common coexisting substances typically present in actual samples. The selected interfering substances include some normal ions (Na^+^, K^+^, Ca^2+^, Mg^2+^, Pb^2+^, Cd^2+^, Fe^3+^, Cl^−^, CO_3_^2−^, SO_4_^2−^, PO_4_^3−^) as well as other OPs (parathion, chlorpyrifos, dimethoate, methanethion, triazophos, metalaxyl, dichlorvos, methamidophos). Under the same experimental conditions, malathion and each interfering substance were added to the probe system at the same concentration, respectively, and the 652 nm absorbance responses were collected and compared simultaneously. As indicated in Figure 7, compared to the effect of malathion, the changes in absorbance values caused by common ions and organic phosphorus pesticides can be ignored. These results indicate that the sensing probe exhibits acceptable anti-interference performance toward malathion under the test conditions set in this study. It should be noted that the molecular recognition and anti-interference mechanisms underlying the selectivity of this sensing system remain to be further elucidated.

### 3.8. Analysis of Malathion in Actual Sample

For the assessment of the real-sample detection capability of the established malathion colorimetric sensing system, this work conducted spiked recovery experiments by adding three gradient concentration levels of malathion standard solution (0.1, 0.5, and 2.0 μg/g) to blank samples of cucumber and cabbage, respectively. As summarized in Table S3, the average recoveries for malathion in cucumber and cabbage samples ranged from 97.2% to 104.5% and 99.4% to 102.5%, respectively, and all relative standard deviations (RSDs) were kept below 7%. The above results demonstrate no significant matrix interference during the analysis. Therefore, based on the results of spiked recovery experiments in this study, the colorimetric method can quantify malathion residues in cucumber and cabbage, which is expected to provide technical support for the rapid detection of malathion residues in food.

## 4. Conclusions

In conclusion, this work successfully synthesized Au@MnO_x_ with excellent OXD-like activity. On this basis, a H_2_O_2_-free colorimetric sensing platform, namely the Au@MnO_x_/TMB/ACP/AA2P system, was developed for malathion detection. The sensing mechanism relies on the redox reaction between the specific hydrolysis product AA of ACP and oxTMB. Coupled with the suppression of ACP activity induced by malathion, a “signal inhibition-recovery” mode regulated by AA and malathion was designed, which achieved high-sensitivity determination of malathion in food matrices. It is worth noting that the detection readout only requires simple optical measurement without large or expensive analytical instruments, and the entire procedure can be completed within 40 min. The developed approach achieved a considerably low LOD value of 1.51 ng/mL, which is superior to the results reported in the previous literature. Therefore, this study provides technical support for the quantitative detection of malathion in food. In addition, the developed malathion colorimetric probe demonstrated satisfactory precision and recovery in actual sample analysis, which met the demands of practical analytical requirements. Overall, this method has significant potential and practical value in the field of food safety, achieving quick and sensitive measurement of malathion.

## Data Availability

The original contributions presented in the study are included in the article/Supplementary Material; further inquiries can be directed to the corresponding author.

## Supplementary Materials

The following supporting information can be downloaded at https://www.mdpi.com/article/10.3390/foods15213967/s1. Figure S1: EDS spectrum and quantitative elemental analysis of Au@MnO_x_.Figure S2: UV-vis absorption spectra of the oxidation of different chromogenic substrates catalyzed by Au@MnO_x_. Experimental conditions: pH 4.0 HAc-NaAc buffer, 3 mM TMB, 100 μg/mL Au@MnO_x_.Figure S3: Effect of buffer solution type on the catalytic oxidation of TMB by Au@MnO_x_.Figure S4: Effect of (A)pH value and (B)concentration of HAc-NaAc buffer on the catalytic oxidation of TMB by Au@MnO_x_.Figure S5: Optimization of (A) TMB concentration and (B) Au@MnO_x_ concentration on the catalytic chromogenic performance.Figure S6: Effect of (A) reaction time and (B) temperature on the catalytic chromogenic performance of Au@MnO_x_ toward TMB.Table S1: Kinetic parameters of the Au@MnO_x_ and other nanozymes catalysts.Table S2: Comparison of analytical performance with other OPs detection methods.Table S3: Recovery and precision of the proposed method for malathion detection in actual food samples.
