# Supplementary material for: Colorimetric Sensor Based on the Oxidase-like Activity of Au@MnOx Nanozyme for High-Sensitivity Detection of Malathion
Source: Foods. 2026 Nov 5;15(21):3967. doi: 10.3390/foods15213967 (PMC13763511; doi:10.3390/foods15213967)
Supplement: Supplementary file 1 [file foods-15-03967-s001.zip › foods-4602137-supplementary.pdf]

Supplementary Materials

Colorimetric sensor based on the oxidase-like activity of $Au@MnO_x$ nanozyme for high-sensitivity detection of malathion

Lu Lin [1, 2 *], Xuan Wang [1], Li-Jiao Wang [1], Guo-Yuan Xiong [1]

[1] College of Food Science and Engineering Anhui Science and Technology University, Chuzhou 239000, China

[2] Anhui Province Key Laboratory of Functional Agriculture and Functional Food, Anhui Science and Technology University, Chuzhou 239000, China

[*] Correspondence: linl@ahstu.edu.cn

Contents:

Figure S1. EDS spectrum and quantitative elemental analysis of Au@MnO_x.

Figure S2. UV-vis absorption spectra of the oxidation of different chromogenic substrates catalyzed by Au@MnO_x. Experimental conditions: pH 4.0 HAc-NaAc buffer, 3 mM TMB, 100 μg/mL Au@MnO_x.

Figure S3. Effect of buffer solution type on the catalytic oxidation of TMB by Au@MnO_x.

Figure S4. Effect of (A) pH value and (B) concentration of HAc-NaAc buffer on the catalytic oxidation of TMB by Au@MnO_x.

Figure S5. Optimization of (A) TMB concentration and (B) Au@MnO_x concentration on the catalytic chromogenic performance.

Figure S6. Effect of (A) reaction time and (B) temperature on the catalytic chromogenic performance of Au@MnO_x toward TMB.

Table S1. Kinetic parameters of the Au@MnO_x and other nanozymes catalysts.

Table S2. Comparison of analytical performance with other OPs detection methods.

Table S3. Recovery and precision of the proposed method for malathion detection in actual food samples.

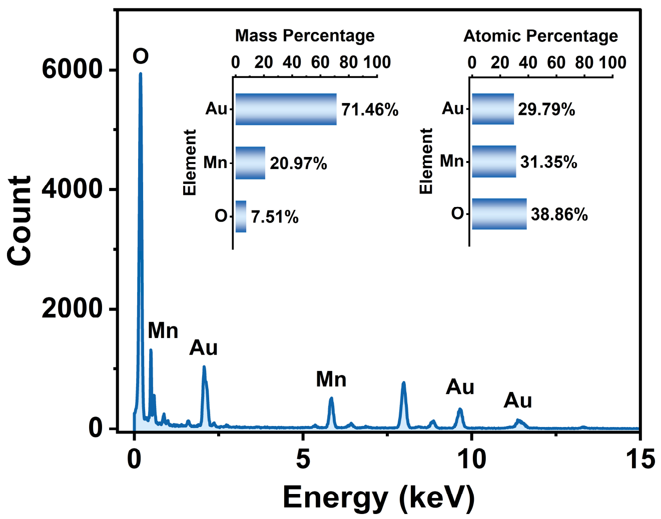

Figure S1. EDS spectrum and quantitative elemental analysis of Au@MnO$_x$.

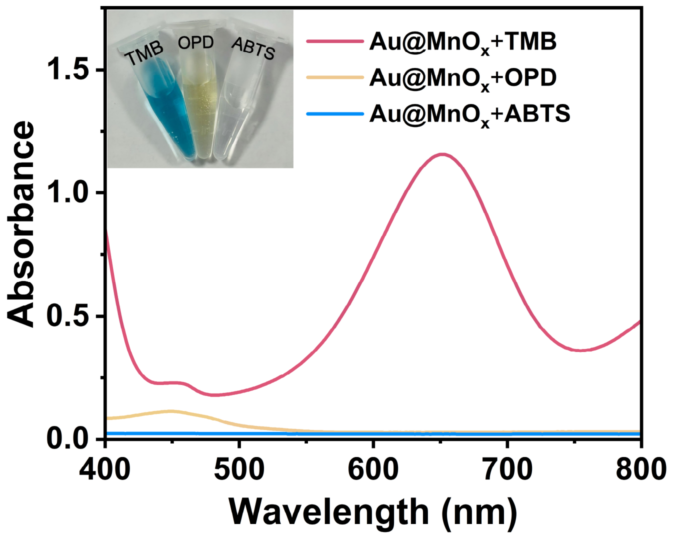

Figure S2. UV-vis absorption spectra of the oxidation of different chromogenic substrates catalyzed by Au@MnO$_x$. Experimental conditions: pH 4.0 HAc-NaAc buffer, 3 mM TMB, 100 μg/mL Au@MnO$_x$.

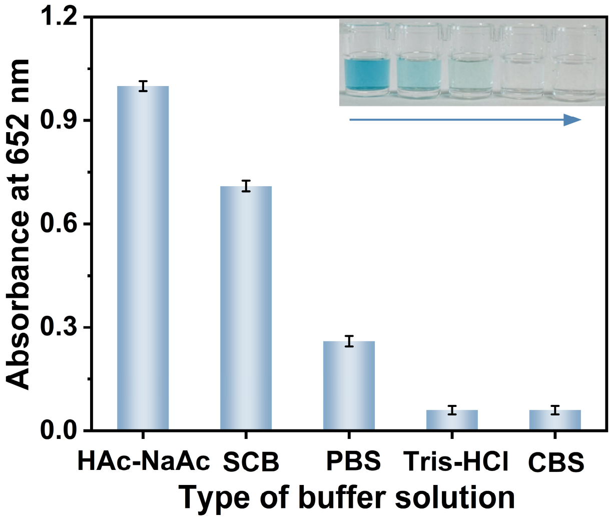

Figure S3. Effect of buffer solution type on the catalytic oxidation of TMB by Au@MnO_x.

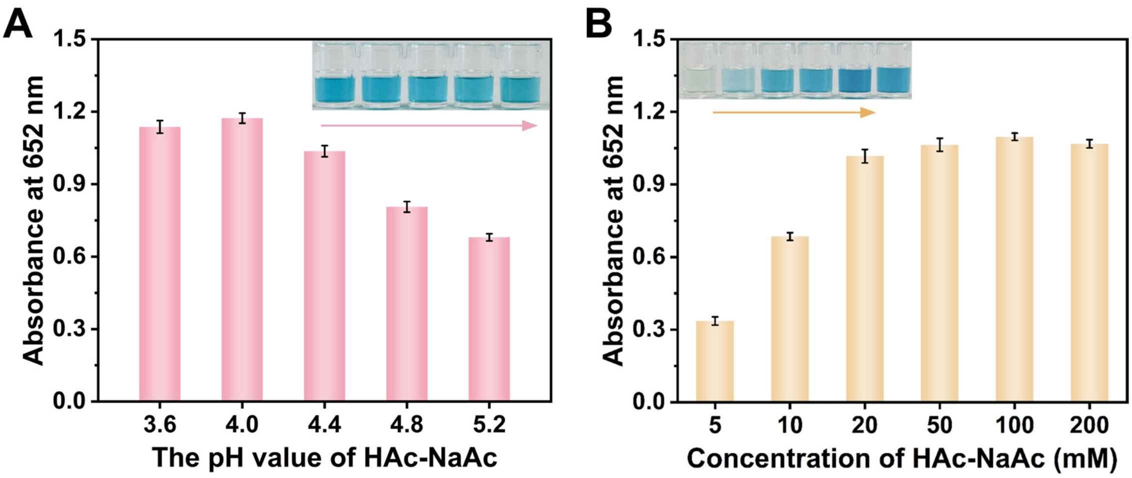

Figure S4. Effect of (A) pH value and (B) concentration of HAc-NaAc buffer on the catalytic oxidation of TMB by Au@MnO_x.

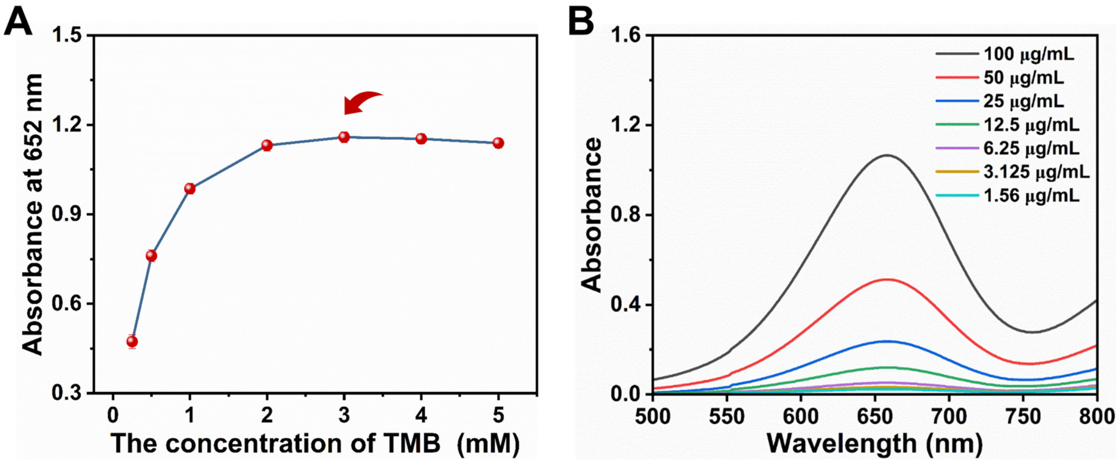

Figure S5. Optimization of (A) TMB concentration and (B) Au@MnO_x concentration on the catalytic chromogenic performance.

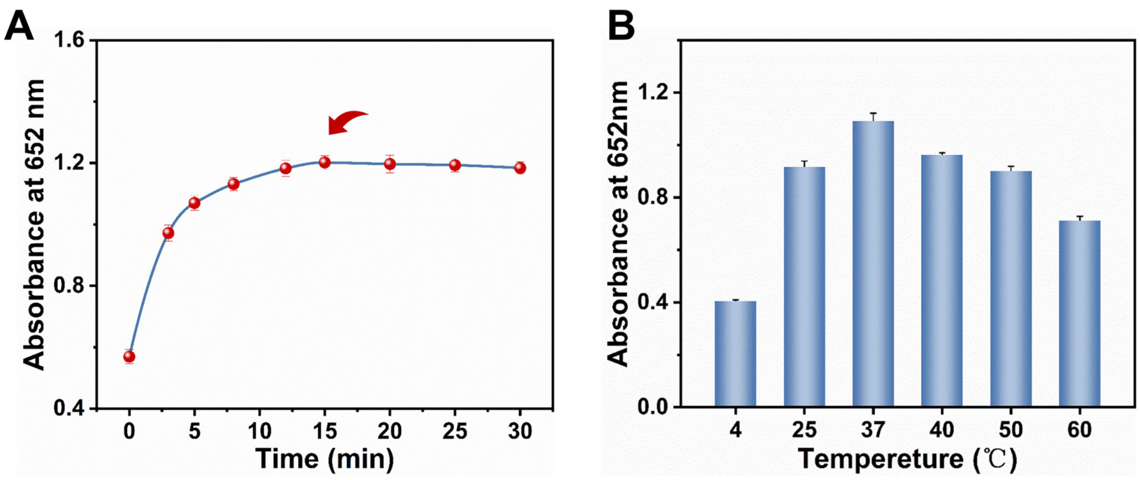

Figure S6. Effect of (A) reaction time and (B) temperature on the catalytic chromogenic performance of Au@MnO_x toward TMB.

Table S1. Kinetic parameters of the Au@MnO_x and other nanozymes catalysts.

Catalyst	K_m (mM)	V_{max} (10^{-8} $M \cdot s^{-1}$)	References
PDA-Pd/NCs	0.211	19.4	[47]
Mn@NC	0.378	18.24	[48]
MCDs@PB	0.56	11.12	[49]
AuPt@Fe-N-C	0.2199	11.05	[30]
Fe_1/SCN	0.067	4.70	[50]
Au@Pt	0.199	7.457	[51]
Au@Pd	0.6366	4.191	
Au@MnO_x	0.146	10.95	This work

Table S2. Comparison of analytical performance with other OPs detection methods.

Materials	Method	Linear range (ng/mL)	LOD (ng/mL)	References
Pt-Ni NPs	Colorimetry	5–2500	1.2	[5]
Fe-MOF	Colorimetry	10–500	5.8	[52]
	Electrochemical	10–500	4.6	
GSH-AuNCs	Fluorescence	10–2000	2.05	[53]
Ce/Zr-MOF@FP	Colorimetry	0.5–500	0.32	[54]
Cu-MOFs	Colorimetry	0–1000	16	[55]
Au@MnO_x	Colorimetry	0–500; 500–3000	1.51	This work

Table S3. Recovery and precision of the proposed method for malathion detection in actual food samples.

Samples	Spiked level (μg/g)	Found value (μg/g)	Recovery (%)	RSD (%, n=3)
Cucumber	0	ND	−	−
	0.1	0.103±0.0067	103.0	6.5
	0.5	0.486±0.0185	97.2	3.8
	2.0	2.09±0.1087	104.5	5.2
Cabbage	0	ND	−	−
	0.1	0.101±0.0059	101.0	5.8
	0.5	0.497±0.0263	99.4	5.3
	2.0	2.05±0.1292	102.5	6.3

ND, not detected; −, values could not be calculated.
